# Types of stigma experienced by patients with mental illness and mental health nurses in Indonesia: a qualitative content analysis

**DOI:** 10.1186/s13033-021-00502-x

**Published:** 2021-10-18

**Authors:** Muhammad Arsyad Subu, Del Fatma Wati, Netrida Netrida, Vetty Priscilla, Jacqueline Maria Dias, Mini Sarah Abraham, Shameran Slewa-Younan, Nabeel Al-Yateem

**Affiliations:** 1 University of Sharjah, Sharjah, UAE; 2 Universitas Binawan, Jakarta, Indonesia; 3 Institute of Health Sciences, Fort De Kock, Indonesia; 4 University of Andalas Padang, Padang, Indonesia; 5 Western Sydney University, Penrith, Australia; 6 School of Nursing, Midwifery and Indigenous Health, Charles Sturt University, Orange, NSW Australia

**Keywords:** Stigma, Mental Health, Indonesia, Qualitative study, Content analysis

## Abstract

**Background:**

Stigma refers to the discrediting, devaluing, and shaming of a person because of characteristics or attributes that they possess. Generally, stigma leads to negative social experiences such as isolation, rejection, marginalization, and discrimination. If related to a health condition such as mental illness, stigma may affect a person’s illness and treatment course, including access to appropriate and professional medical treatment. Stigma has also been reported to affect patients’ families or relatives, along with professionals who work in mental healthcare settings. Stigma is strongly influenced by cultural and contextual value systems that differ over time and across contexts. However, limited information is available on how types of stigma are experienced by patients with mental illness and mental health nurses in Indonesia.

**Method:**

We explored the stigma-related experiences of 15 nurses and 15 patients in Indonesia. The study design and analysis of interview data were guided by deductive (directed) content analysis.

**Results:**

Five themes emerged. Four themes were patient-related: personal/patients’ stigma, public/social stigma, family stigma, and employment stigma. The fifth theme related to stigma toward healthcare professionals working with patients with mental illnesses, which we categorized as professional stigma.

**Conclusions:**

This study has achieved a deep understanding of the concept of stigma in the Indonesian context. This understanding is a prerequisite for developing appropriate interventions that address this phenomenon and thereby for the development of mental health services in Indonesia. This study may also be transferable to other countries that share similar cultural backgrounds and adhere to traditional and religious value systems.

## Background

Stigma was initially described by Erving Goffman in 1963. He identified stigma as any characteristic or attribute by which a person was devalued, tainted, or considered shameful or discredited. Subsequent work in this area was influenced by the work of Goffman, and the concept of stigma has been explored in many contexts and cultures. Stigma is strongly influenced by cultural and contextual value systems that differ over time and across contexts. However, most authors agree with Goffman’s basic definition, which identified the main elements of stigma such as labeling, stereotyping, social isolation, prejudice, rejection, ignorance, status loss, low self-esteem, low self-efficacy, marginalization, and discrimination [1–3].

Mental health stigma is defined as the disgrace, social disapproval, or social discrediting of individuals with a mental health problem [4, 5]. Literature identifies multiple dimensions or types of mental health-related stigma, including self-stigma, public stigma, professional stigma, and institutional stigma. Self-stigma refers to negative attitudes of an individual to his/her own mental illness and is also referred to as internalized stigma [1, 6]. Self-stigma has been related to poor outcomes, such as failure to access treatment, disempowerment, reduced self-efficacy, and decreased quality of life [7, 8]. Public stigma refers to negative attitudes towards those with mental illness by held by the general public [1, 6], often based on misconceptions, fear, and prejudice. Related to public stigma is perceived stigma which is defined as individual’s beliefs about the attitudes of others towards mental illness. Research has demonstrated the significant impact of public stigma such as discrimination in workplaces and public agencies [8]. Professional stigma occurs when healthcare professionals hold stigmatizing attitudes toward their patients, which are often based on fear or misunderstandings of the causes and symptoms of mental illness, or when professionals themselves experience stigma from the public or other healthcare professionals because of their work and connection with stigmatized individuals [1]. Professional stigma is of particular concern as it may affect the care and treatment a person with mental illness receives [1], including treatment for physical illnesses [8], thereby impacting their well-being and recovery. Finally, institutional stigma refers to an organization’s policies or culture of negative attitudes and beliefs toward stigmatized individuals, such as those with mental health problems [1, 6–14]. Such stigma can also be reinforced by legal frameworks, public policy, and professional practices, thereby becoming deeply embedded in society [8].

In the context of mental healthcare, stigma has been identified as a major issue for patients and families. Stigma hinders access to appropriate and professional medical and psychological treatment, and can result in a person’s condition worsening or multiple readmissions [3, 6, 7, 15]. Furthermore, the impact of stigma is so great that patients describe the stigma and prejudice they encounter as almost as bad as the symptoms of their disorder [16], and as a burden on their private and public lives [17]. Stigma also affects patients’ families or relatives and the professionals who work in mental healthcare settings. Therefore, to reduce stigma in mental healthcare and facilitate the development of appropriate services in Indonesia and similar countries or contexts, it is important that the different types of stigma are clarified and understood within the unique value system and culture.

The Republic of Indonesia has the fourth largest population in the world and the third largest in the Asian continent. As estimated in 2020, the Indonesian population comprises 267 million people; approximately 151 million people (around 56.6% of the Indonesian population) live in urban areas and the remainder lives in rural areas [18, 19]. In general, Indonesians follow a traditional way of life that is strongly affected by traditional and religious beliefs. The prevalence of severe mental illness in Indonesia is estimated at 1.7/1000 population, and that of mild mental illness is around 60/1000 population [18]. Stigma is known to be common in such traditional contexts [19, 20]. Therefore, understanding how stigma manifests in this context will help reduce stigma and contribute to developing mental healthcare services in Indonesia and potentially in other similar Asian contexts.

Treatment for mental illness in Indonesia is currently inadequate. The country has the lowest ratio of psychiatrists per capita in the world, and mental healthcare facilities are limited in availability and underdeveloped in terms of quality, human resources, and infrastructure [20–22]. This situation, along with low public awareness of mental illness, persisting stigmatizing and traditional beliefs about mental health, and the lack of local professional knowledge in the area, seriously impact the care of patients with mental illness in terms of access to and quality of services. In addition, stigma about mental illness is rarely discussed openly, which results in misunderstanding, prejudice, confusion, and fear. In this context, families often hide or ostracize family members with mental illness because they are reluctant to bring them to public attention or seek help [18, 21, 23].

A recent study found that the experience of stigma among patients with mental illness in Indonesia was pervasive and negatively impacted use of mental health services[24]. The stigmatization of mental illness is manifested by families, community members, mental health professionals and staff, governmental institutions, and the media. Stigmatization is characterized by violence, fear, exclusion, isolation, rejection, blame, discrimination, and devaluation, primarily as a result of general (mis)understandings about mental illness. Until the stigma associated with mental illness is addressed at the national level, Indonesians with mental illness will continue to suffer and face barriers to accessing mental health services [24]. Given Indonesia’s predominantly rural population and traditional way of life, it is particularly important to examine stigma in this context. For example, persisting stigma means that families in traditional societies such as Indonesia and other Asian countries hide those with mental illness because of embarrassment and shame, and are unwilling to access public mental health services [25]. Stigma may also prevent a family from socializing with other community members. In addition, others may blame family members for the person’s illness, meaning patients experience further feelings of shame and guilt [24–28]. It has also been reported that stigma means that health professionals in psychiatric hospitals often do not treat patients with dignity or respect, and do not provide optimal protection for patients who are hospitalized [29].

Despite the prevalence of mental illness and the high levels of stigma toward patients with mental illness, little research has been conducted to clarify the elements, attributes, and features of different types of stigma in the Indonesian culture and value system. A literature review on mental health in Indonesia conducted in PubMed returned 161 studies published between 1949 and 2020. However, only 15 of these studies discussed stigma either directly or in the context of Indonesian mental health services [18, 30–43]. Among these 15 studies, six discussed stigma in general, consequences of stigma (i.e., “pasung” or confinement), attitudes toward mental health, and perceptions of mental health [18, 30, 31, 41–43]. Previous investigations of stigma in Indonesia mainly examined personal stigma, with a focus on the perceptions of those with mental illness and their families, how they respond to stigmatization in their lives, and the impact of stigma on access to mental health facilities or treatment [18, 20–23, 44]. The present study offers a unique perspective given its comprehensive approach to understanding the different types of stigma that exist in Indonesian culture.

## Question guiding this inquiry

This qualitative study explored different types of stigma that affect individuals with mental health conditions in Indonesia, as described by patients and nursing staff. Specifically, we aimed to clarify the elements, attributes, and features of different types of stigma experienced by patients and nursing staff in Indonesia. Exploring these diverse perspectives allowed us to achieve an in-depth understanding of stigma in this context. The results of this study therefore build on existing literature and may inform specific and effective interventions targeting different types of stigma. Finally, given Indonesia’s size and the similarity of its cultural context to other Asian countries, the results of this study may improve our understanding of types of stigma experienced in the wider Asian context.

## Methods

### Study design

This study used a qualitative design based on deductive (directed) content analysis. Content analysis is considered a research method or technique as well as a data analysis tool [45, 46]. Research using deductive (directed) content analysis usually has prior theoretical knowledge as the starting point [47], and this study was informed by previous research findings and theories focused on mental health stigma. Bengtsson (2016) outlined the different stages of qualitative content analysis (e.g., identifying the study problem, planning data collection methods, and data analysis) and described both “manifest” and “latent” analysis techniques. The present study used a latent analysis technique, whereby we attempted to understand underlying meanings reflected in the data in the context of extant knowledge and theories relevant to the topic under study. This aided in developing a deeper understanding of individual meanings and experiences of stigma in this context. Using deductive (directed) content analysis allowed us to explore meanings related to mental illness stigma constructed by patients with mental illness and nurses, and describe and interpret those meanings. In addition, this study design allowed us to go deep into the data to reveal participants’ thoughts and experiences that were close to their realities at that particular place and time.

### Study setting and participants

This study was conducted at the largest of Indonesia’s 33 psychiatric hospitals. The hospital is located in West Java, which is one of the 34 provinces in Indonesia, and receives patients referred from across the province (urban and rural areas). West Java is an ethnically diverse province with a range of inhabitants from various ethnicities. Study participants were 15 patients (seven males, eight females) and 15 psychiatric nurses (10 males, five females) recruited purposefully from the hospital. Although all participants were recruited in the same site, they originated from both urban and rural areas. The majority of participants were Sundanese, and some were Javanese. Patient participants were aged 21–52 years with mild/moderate symptoms (as noted in their medical records). Nurse participants were psychiatric nurses who had graduated from nursing schools with a specialty in mental health nursing. These nurses were aged 22–43 years and had 5–15 years of clinical experience in mental health settings. We based our sample size (N = 30) on a previous study [48], which indicated that 30 participants was sufficient to ensure data saturation.

Our initial contact with participating nurses was made through a meeting and a presentation about this study at the study hospital. This initial meeting was followed by data collection at a mutually agreed time. We made contact with patient participants after discussion with the hospital healthcare team. We excluded patients with severe psychosis or severe symptoms of their mental illness. All participants were required to be able to read and write. Data were collected at the hospital through interviews held in environments that were private and quiet to facilitate participants’ comfort and confidence to speak. All participants signed an informed consent form before their interview and were assured of the anonymity and confidentiality of their data. All data were coded for analysis, and all data (including field notes and memos) were kept securely by the primary investigator.

### Data collection

The primary method of data collection was semi-structured interviews. Interview questions were developed based on themes identified during a literature review. Interview questions were not prescriptive, but were used as a guide to explore aspects that were considered vital to understand the elements of the different types of mental health-related stigma and stigmatization experienced by participants in traditional Indonesian culture and reality. Interviews took from 30 to 45 min for both nurses and patients’ participants. Interview questions were phrased to suit participants (i.e. nurses and patients). Sample of interview questions is below:

For patients:

- In your opinion how does Indonesian society see or deal with mentally ill persons?
- How is it to live in Indonesia when you have a mental health issue?

For nurses:

- In your opinion how does Indonesian society see or deal with mentally ill persons?
- How is it to live in Indonesia for someone who have a mental health issues?
- Could you give me an idea about your work in a mental health hospital?

Additional data were collected via memos, field notes, and a document review. These additional data collection methods enabled data triangulation, which improved the credibility of the interpretations of the data. The interviewer also used memos to record their thoughts and interpretations of the interviews, the research process (including questions and gaps), and the analytic progress of the research. Field notes were used to record observations and reflections on the data. We also conducted a document review to collect hard copy and electronic data that were available in the hospital. This mute evidence was important in guiding our interpretation of participants’ experiences, attitudes, and beliefs.

In total, we conducted 30 semi-structured interviews in the Indonesian language (Bahasa Indonesia). To ensure the interviews were consistent, all interviews were conducted by two experienced interviewers (MAS and DFW), who were local members of the research team. At the beginning of the interview with each participant, the interviewers introduced themselves and explained the purpose of the study and the confidential nature of the data collected. This gave participants opportunity to ask any questions and helped to establish a comfort level before the interview began. The appropriateness of the interview location and timing was verified with participants; the interviewers tried not to take up too much of their time, and were prepared to provide emotional support to participants when necessary. Before their interview started, each participant confirmed that they had read the participant information sheet and were fully informed about the study. The informed consent process was completed before the start of the interview (in Bahasa Indonesia).

All 30 participants attended on the scheduled day of their interview. The interviews were conducted in hospital meeting rooms or nurses’ offices. The questions were asked in the order they were presented in the interview protocol. During the interviews, participants were given time to reflect on and consider their responses to ensure they did not feel pressured to respond before they were ready. Participants were given opportunity to ask more questions at the end of the interview. Finally, the interviewers expressed gratitude for participants’ time and willingness to participate in this study. Immediately after the interview, the information was summarized, and field notes and memos were checked.

### Data analysis

We analyzed data using deductive (directed) content analysis. This method was suitable for this study as we aimed to gain a deep understanding of the experiences of Indonesian patients with mental illness and mental health nurses in relation to the different types and categories of mental health stigma reported in the literature. The interviews were analyzed by the Indonesian members of the research team, who then translated important quotations into English for reporting. Linguistic equivalence was an important consideration during the translation process to ensure the integrity of our findings. The first author (MAS) has English as his first language. During this translation process, this author (MAS) was assisted by an Indonesian professional English nursing translator to ensure linguistic equivalence. The translation process focused on verifying that the translation from Indonesian to English was correct in terms of words, terms, concepts, and overall meaning. This ensured that the English translations were comprehensible, but faithful to the interview data obtained from participants.

During the analysis process, we read the interview transcripts several times to become familiar with the text. Next, we merged and coded the words, sentences, and paragraphs line-by-line, as relevant to each other in terms of both the content and context of stigma. Then, parts related to the experiences of the participating patients and nurses regarding types of stigma were extracted and placed in a separate text file. Codes and units of meaning were interpreted in the context of the study and compared in terms of similarities and differences. Finally, abstract themes were developed reflecting types of stigma consistent with the literature.

## Results

Study participants were 15 patients with mental illness (seven men, eight women) who were hospitalized in the participating psychiatric hospital, and 15 nurses (five women, 10 men) who worked in the same hospital. Our analysis revealed five main themes. Four themes were related to patients with mental illnesses and loosely classified under the categories of either public or perceived stigma. These themes were ‘perceived stigma from a patient perspective’, ‘public stigma’, family ‘attitudes’, and ‘employment discrimination’. The fifth theme, professional stigma, described stigma experienced (or held) by healthcare professionals who worked with patients with mental illnesses. Although these themes reflected the stigma experienced by participants in our study, they were consistent with the types of stigma described in the literature. This was because we used deductive (directed) content analysis, which draws on existing knowledge and theories as the starting point for the analysis.

### Theme 1: perceived stigma from a patient perspective

The theme of personal/patients’ stigma was strongly represented in the narratives of all participating patients, but received little attention from participating nurses. Therefore, only findings related to patients’ perspectives are presented. Participants described feelings of shame and isolation from the community, and indicated that they were viewed as different from other “normal” people. They also believed that others thought they were inadequate, and reported suffering insults. A patient described being insulted as making them ashamed:

*Within society, there is insult, discredit…They insult me. I am called “crazy,” or “former crazy people.” Yes, I have been insulted. It is from friends and the community too. I cannot do anything. I am sad and ashamed, my heart cries without tears. I hope that God helps me... (Participant 4)*

Participants also indicated that they were labeled as a “mentally ill person.” One patient reported that this labeling was part of their suffering.

*If in the community, people see me [they say], “whew a professor’s patient.” People say “wuhh wuhh” (see lowly), “the patient of doctor R, wuhhh (low).” Yes, they do look down. If I visit my professor, my label is not for recovery. Moreover, they make a label for me as the professor’s patient; it means that I am a mentally ill person. They consider me like that… (Participant 7)*

Participating patients also believed that they were rejected, avoided, and discriminated against because they had a mental illness. Further, they reported that community members rejected them because society held wrong assumptions about mental illness. One participant reported:

*Yes, they (patients) are rejected, like that. They actually can be accepted again by their community, but they (the community) don’t accept. I also need to change. So, we are “crazy” depend on us. There are still wrong assumptions in the community about mentally ill people. Yes...probably, only few of them, I see, who can be accepting. (Participant 2)*

Another participant described how they had been discriminated against by others.

*…All the problems are because the problem cannot be solved by our family members. I experience that there is discrimination…Other people treat us as unequal with people who have physical illness. Yes, they [general people] do discriminate… (Participant 5)*

### Theme 2: public stigma

Public/social stigma was an issue highlighted by both groups of participants. Therefore, this theme is discussed from both patients’ and nurses’ perspectives.

#### Patients’ perspectives

Support from community members or other social support is essential for improving outcomes for people with mental illness. However, most patients felt they lacked this social support.

*…I do not have support…I don’t have support at all. I do not know why it is like that sir. My community, my sister and other people do not provide support, no [support]. They only care [about] themselves. No, I do not have support from other people when in this hospital. They just want me to go to witchcraft, go to a shaman. (Participant 11)*

Of particular concern, some participants reported forms of community violence towards those with mental illness. Participating patients described experiencing violence from people in the community. One patient shared their experience of being hit and tied by other people in their community.

*…Yes, I was tied and hit. Therefore, I was really angry and I don’t want to meet the people in my community. However, if they come to my home and apologize to me, then, I will come to apologize at their homes. Therefore, I still feel [the need for] revenge because I was tied and hit. (Participant 1)*

Additionally, participants perceived that many people in the community believed that mental illness is a communicable illness, similar to some physical illnesses.

*...They stay away from sufferers. Sometimes, they are afraid that mental illness is a contagious illness. People are afraid because of this. Actually, sufferer isn’t harmful. If they are embraced, they will be OK. (Participant 5)*

#### Nurses’ perspectives

Some nurse participants noted that the general public/wider society lacked consideration and empathy toward those who suffered from mental illness. This lack of consideration and corresponding lack of appropriate policies often resulted in homelessness and isolation among people with mental illness.

*Our societies still lack care...less attention for people with mental illness. Because communities lack care, communities do not care. They just ignore…I am sure, if 10 people [met a person] with mental illness, only one or two people will still want to say hello or interact with them. (Nurse 7)*

A lack of social acceptance was reported by participants as resulting in people with mental illness being rejected. A nurse described the impact of patients not being accepted in their community.

*Because they (patients) could not be accepted in society…society cannot accept them. Other people reject them. In addition, their families cannot be accepted by society. Patients cannot be productive anymore; minimal in fulfilling their basic needs. (Nurse 1)*

In general, participants reported that community members feared dangers posed by those with mental illness. Participants indicated that people were afraid of patients with mental illness because of a perceived tendency for violent behavior or fear of being attacked by a patient. A nurse described how people were often scared and ran away.

*They (community members) are afraid, sir…afraid…scared…anxious. The society is scared. Yes, it is really, true [people] are scared, run away, they are like that. They (patients) are ignored, they are left, finally [laughs]. Because they (society) are scared, the patients are ignored. (Nurse 1)*

### Theme 3: family attitudes towards mentally ill patients

Similar to public/social stigma, both participant groups shared their views and experiences of family stigma. Therefore, findings reflecting both patients’ and nurses’ perspectives are presented.

#### Patients’ perspectives

Family support plays an important role in the recovery of a person with mental illness. However, our participants indicated that their family members provided minimal support because of stigma and shame.

*They (family members) do not talk to me. They do not support me. Sometimes, my parents are ashamed…My father is not proud of me in front of other people. For example, “my son is like this.” “See! My son has been like this.” What can he tell others? Other parents will say “my child goes to the college in Jayabaya (a university) takes informatics engineering field.” My parents do not mention about me like that. My father and mother do not do that…They are ashamed. (Participant 10)*

#### Nurses’ perspectives

Participating nursing staff described how many families had moved to another location or changed their address because of feelings of shame. They indicated that some families also denied they had relatives who were treated in the hospital.

*Their families disappear because of shame? The first, their domicile (address) changes. Then, although we go there and we find the address, they say “I have no family relationship with him, all his family members have died.” Some patients have been here since they were young. Probably, they are the patient’s family. People who were at home are his family members, but they don’t acknowledge him. “We don’t recognize that patient” [the] family said. (Nurse 8)*

Participants also indicated that the extended families often reject relatives that suffered from mental illness. This rejection was reported to happen even after a patient’s hospitalization.

*Especially for long-time patients, for example, [those who have spent] many years here, it is difficult to get them (families) to take their relatives home. Because they assume that at home, the patient will annoy them. They will annoy their family’s activities. Then, [the] patient is rejected. Mostly they are rejected… (Nurse 6)*

### Theme 4: Employment discrimination

Both nurses and patients commented on the stigma that people with mental illness experience in the context of their employment. The perspectives of both nurses and patients are presented below. Overall, most participants indicated there was a great deal of stigma related to mental illness in workplaces.

#### Patients’ perspectives

Returning to work after treatment was reported as difficult. Many patients were rejected from returning to their former workplace, including a patient that was previously employed in a government role.

*…Yes, it is, very often. Other people do not want to accept a patient to work again in his job…“You are an ex-crazy individual.” They will say that. I was a government employee…The government officers will not allow me to work again. (Participant 2)*

#### Nurses’ perspectives

A nurse described how patients found it difficult to find a job because of having a mental illness label.

*…If they have mental hospital label or have stigma from society, then looking for a job, it is difficult for them. I have had a house assistant who has been violent. Then, she worked at my parents-in-law; I was worried to leave her at home alone. I asked: “did you hear voices?” She said “yes.” I was scared too. Fortunately, she asked to resign. (Nurse 2)*

### Theme 5: professional stigma

The fifth theme that emerged from the interviews was professional stigma. Two forms of professional stigma emerged in this study: stigma directed toward mental health nurses, and stigma from healthcare professionals toward their patients with mental illness. Although this type of stigma was mostly present in nurses’ narratives, some patients reported experiencing stigma from healthcare professionals; therefore, both nurses’ and patients’ perspectives are discussed.

#### Nurses’ perspectives

Participating nurses described how mental health nurses were labeled as “crazy nurses,” which captured the stigma directed to nurses working with patients with mental illness. Some nurses shared examples of how non-mental health nurse colleagues and the general public used terms that insulted them.

*…People say “ohhhhh [it is] because you are psychiatric nurse”...“Uhhhhh...yeah, since he works to care for the gelo (crazy) people.” They say “whew psychiatric nurse.” Sometimes this stigma sticks to the nurses from people in our society. Yes…also, we are labeled by our friends (nurses). They are either joking or serious, I do not know. In addition, my friends say: “ihhhhh...whew, psychiatric nurse.” Yes, similar, as crazy as his patients. (Nurses 6)*

It was also noted that as well as the general public, some healthcare professionals also believed that mental illness was contagious, similar to many physical illnesses.

*….Yesterday, there was a student who feared to be contaminated by this illness. She is a student from Palangkaraya, Kalimantan who fears of contamination too. Besides fearing being contaminated, they [are] disgusted [by] mentally ill patients. Their disgust with mentally ill people is similar to leprous, dirty, disgusting. In reality, mental illness does not spread, right? Then, there is an image (label)...“Don’t have relation[ships] with the patients.” (Nurse 4)*

#### Patients’ perspectives

Participating patients also reported that some healthcare professionals held stigma toward patients with mental illness. They noted this stigma was frequently manifested in the use of restraint or seclusion. Some nurses and other hospital staff were reported to physically abuse their patients.

*Yes, I was tied. True, it was true. I have to tell you. My jaws are tied, by Mr. A (a nurse). I was injected, it was pain, right? I don’t understand, probably his education wasn’t high enough so that he didn’t understand. My need isn’t food, but, they just don’t care… (Patient 9)*

## Discussion

Stigma has significant impacts on patients with mental illness, family members, communities, and healthcare professionals. To date, little research has investigated the types of stigma and corresponding impact in the Indonesian context. Stigma is a worldwide concern that influences people’s illness trajectory, treatment process, available opportunities, quality of life, and recovery outcomes. Our study sought to investigate the types of stigma experienced by people with mental illness and mental health professionals in the Indonesian context. Using a deductive approach (directed content analysis), our findings on the examples of stigma reported were loosely centred around public and perceived stigma, consistent with the types of stigma previously reported in the literature [1, 6–14]. However, we identified features of these types of stigma in the Indonesian context, and our study therefore makes a direct contribution to the literature. As described in previous studies [7, 8], stigma is a burden for patients with mental illness that can be intrapersonal (self-stigma), interpersonal or in relationships with other people, and structural or discriminatory stigma relating to exclusionary policies and other aspects of life or systems. Our participants shared their experiences of different types of stigma, along with the corresponding elements, attributes, and features within the unique Indonesian culture and value system.

### Perceived stigma from a patient perspective

Many participants described feelings of shame and being rejected and isolated from society, which resulted in feelings of powerlessness. A plausible explanation for this is that patients had internalized stigma (self-stigma) because of negative attitudes and beliefs toward them. Other studies reported that around 40% of people with severe mental illness had high levels of self-stigma [11, 13]. Self-stigma exists when an individual believes negative stereotypes about mental illness and people with mental illness, and feels that these stereotypes apply to them [14]. In addition, almost 70% of patients reported moderate to high levels of perceived discrimination, which has been significantly associated with high self-stigma [11]. Implicit self-stigma appears to be associated with negative outcomes. It has been noted that patients who internalize stigma do not respond as well to evidence-based interventions as those that do not internalize stigma [14]. Self-stigma has been associated with poor self-esteem, hopelessness, reduced self-efficacy, and disempowerment [9].

Furthermore, our participants’ descriptions of these negative feelings indicated they were implicit processes. This means that they were activated automatically and occurred whether or not the individual deliberately endorsed the proposition that mental illness is shameful. For example, a patient with mental illness or healthcare professionals working with that patient may explicitly disapprove of such stigmatization, but implicitly, they may still experience the shame associated with this stigmatization. Attempts to reduce self-stigma should therefore consider these implicit processes [49]. In addition, our findings indicated that many patients were labeled by others as mentally ill. Participating patients expressed feelings of shame, and spoke about how the label of being a “crazy” person made them feel useless and powerless. For patients, hospitalization in a psychiatric hospital can be experienced as disempowering and stigmatizing [15]. Because self-stigma can have negative effects on an individual’s life and treatment outcomes, it is important for clinicians to be aware of the existence of self-stigma, so they can recognize patients’ internalized stigma and address this effectively in treatment.

### Public stigma

Public stigma has negative effects on the lives of people with mental illness, and creates barriers to the individual’s pursuit of vocational, housing, and healthcare goals [50]. In addition, public stigma affects living, working, and socializing for people with a mental illness [51]. A similar study found that nine out of 10 patients with mental illness had experienced discrimination [52]. Our study also found that both nurse and patient participants reported that people in the community enacted violence toward people with mental illness. For example, because community members were ashamed and afraid of those with mental illness, they commonly subjected people with mental illness to confinement or “pasung” and seclusion or “seklusi” [19]. Confinement/pasung and seclusion/seklusi have a negative impact on patients with mental illness, and their use has potential to cause physical harm and further psychological trauma [29]. In addition, many Indonesians adhere to traditional causal beliefs of mental illness, and these beliefs may drive mental health stigma [21, 22, 26, 34, 37]. Given that Indonesia is a developing country, it is likely that these traditional beliefs underling mental health stigma are common across rural and urban communities.

### Family attitudes

Our findings indicated that stigma related to mental illness also impacted patients’ families. This was consistent with Nurjannah et al. [46], who noted that mental health stigma has negative implications on the health and wellbeing of patients and their families. Various impacts on the families of people with mental illness have been documented, including sleep disorders, alterations in interpersonal relationships, worsening of wellbeing, and reduced quality of life [53, 54]. Further, it has been reported that some families with a relative suffering from mental illness experience shame because other people blame them for being responsible for the illness [24]. A family can also feel ashamed if people in their community know that they have relative with mental illness. Three stereotypes associated with family stigma have been described: shame, blame, and contamination [28]. Our study showed that parents were blamed for their offspring having a mental illness, leading to feelings of shame. It was also noted that family members stayed away from patients when they were in hospital and would not visit them. If nurses conducted home visits, family members denied that they had hospitalized relatives. In addition, we found that family members sometimes perpetrated violence towards relatives who had mental illness. For example, participating patients indicated that families subjected them to pasung/confinement or isolated them in a room (seclusion) because community members ordered them to do so, or because they were ashamed or afraid that the patient would be violent [19, 20].

### Professional stigma

Another significant finding that emerged from participants’ narratives was the presence of professional stigma. This type of stigma included stigma that nurses held toward patients with mental illness, as well as their experiences of being stigmatized by others because of their job. Our participants reported that some nurses and hospital staff held stigmatized attitudes towards patients, which was consistent with existing literature. This type of stigma is a major concern for healthcare professionals, especially nurses, as it may result in disparities in healthcare access and treatment, and affect outcomes [55]. A previous study indicated that despite healthcare professionals’ attitudes towards mental illness being more positive than those of the general public, paternalistic or negative attitudes were also common, especially around prognosis and the (supposed) limited possibilities for recovery of people with mental illness [56]. It may also be that nurses and other healthcare professionals continue to misunderstand the causes and symptoms of mental illness, despite considerable experience in this setting. Nurses may also fear mental illness, especially if they believe that mental illness is contagious and can be transmitted like a contagious physical illness [24]. We speculated that such fear and misunderstanding may be a particular challenge in strongly traditional societies such as Indonesia. Our results also showed that nurses felt that they were discriminated against by other (non-mental health) nurses because they worked in a mental hospital. They also felt humiliated when other people called them as “crazy” as their patients. In addition, our findings indicated that nurses and hospital staff used restraint and seclusion because of fear. This was consistent with a previous study that reported staff may use restraint and seclusion when patients are perceived as dangerous [57].

### Employment discrimination

Having a secure job provides an individual with status in society. For a patient with mental illness, employment is an important part of recovery. Our findings indicated that patients had experienced stigma in their workplaces, which was consistent with the concept of institutional stigma previously reported. The reports of participants in this study suggested that many employers in Indonesia still have negative attitudes and discriminate against people with a mental illness. Many participants reported being refused work because they had a mental illness. In addition, some were not accepted back to their previous place of employment. A previous study found that employment rates for people with severe mental illness were as low as 4% [10]. Discrimination and stigmatizing beliefs and attitudes make it difficult for people with mental illness to find employment [58]. A study involving people living with schizophrenia found that over one-third anticipated discrimination in job-seeking [11]. Stigma can result in difficulties for people with mental illness entering the competitive workforce. Some employers explicitly express negative attitudes regarding workers with mental illness and may be hesitant to hire them [59]. Having a mental illness may also limit a person’s career advancement, as employers are less likely to offer promotion to this group [60]. In addition, people with mental illness reported being passed over for jobs for which they were qualified or fired because of their illness [60]. Our findings showed that although these studies were conducted some time ago, similar issues continue to be experienced by people with mental illness.

## Linking them all together

The result of the study confirms that stigmatization of mental illness within the Indonesian context, similar to other countries is a complex and multiple dimensional phenomenon impacting individuals, families, organizations and within the whole society. The outcomes of different types of stigma include social disgrace at personal and family level, separation and loss of social integration amongst families, friends and relatives, status loss and discrimination, homelessness, unemployment, and treatment avoidance. Professional stigma held by nurse professionals toward mental patients develops very much in the same way as public stigma. Nurses themselves are also the recipients of stigma because their work and their workplace are seen as dangerous or even contagious.

## Study limitations

Stigma may limit patients’ ability to fully disclose their feelings and stigmatization experiences. Therefore, a major limitation of this study was that despite assuring all participants of the anonymity and confidentiality of their data, some participants might not have felt comfortable in expressing or discussing difficult experiences, and might not have been fully open and honest in sharing their perceptions or experiences. Therefore, during the interviews, participants might not have completely disclosed their situation in their responses or withheld information. The small sample size used in this study (N = 30) may also be considered a limitation. However, after our analysis of the interviews transcripts, the research team believed that saturation had been reached and no further data collection was necessary. Another major limitation was that this study relied on the researchers’ interpretation of the meaning implied in the interview data, which means that some bias persists. To minimize this bias, triangulation was performed using several methods of data collection (interviews, field notes, and writing memos). In addition, bias might have been introduced in our sample as all participants were recruited from a single large referral hospital. This limits the generalizability of our findings as participants cannot be considered representative of the entire spectrum of mental health settings in Indonesia. Finally, verification of data analysis and interpretation with participants could have been a useful step to further deepen understanding and increase credibility, this step was not feasible and could not be performed within this study.

Despite these limitations, we believe that our findings are particularly relevant for mental health professionals, as well as for professionals in other fields where patients and families may be exposed to different kinds of stigmatization. The results of this study may be used to inform research activity in similar settings elsewhere, and contribute to improving practice, education, and research in mental health in Indonesia and similar areas. Although generalization is not the intent of qualitative studies per se, it is possible for our results to be considered in the context of other communities or countries with similar cultural and religious backgrounds. The replication of this study in other mental health settings with different groups of participants may produce different important data.

## Conclusion

In Indonesia and other countries with similar cultural contexts, people adhere to traditional and religious value systems that affect (positively or negatively) various aspects of life, including psychosocial aspects and health. Some of these values have strong roots, and therefore have more effects on vulnerable people, which is of particular importance. Traditional beliefs about causes of and treatments for mental illness are prominent in Indonesian culture and traditions, and have seeded the persistent existing taboos or stigma that affect the lives and health of patients, families, and relatives. This study achieved a deep understanding of the concept of stigma in the context of mental healthcare in Indonesia; this understanding is a prerequisite for developing appropriate interventions and the development of mental healthcare services in the country.

## Data Availability

The datasets used and/or analysed in the present study are available from author MS on reasonable request.
